# Presentation and Management of a Novel Ehlers-Danlos COL5A1 Variant With Birt-Hogg-Dube Syndrome: A Case Study

**DOI:** 10.7759/cureus.35866

**Published:** 2023-03-07

**Authors:** Avery N Love, Bruce Palmer

**Affiliations:** 1 Osteopathic Medicine, Andrew Taylor (A.T. Still University School of Osteopathic Medicine, Mesa, USA; 2 Interventional Cardiology, United Regional, Wichita Falls, USA

**Keywords:** case report, birt-hogg-dube, flcn, col, aortic aneurysm, ehlers-danlos

## Abstract

Ehlers-Danlos syndrome (EDS) is a hereditary disorder caused by a mutation in the COL gene, which leads to the faulty synthesis of the collagen protein. EDS can present with a wide array of manifestations depending upon which COL gene is mutated. Birt-Hogg-Dubé (BHD) syndrome is a rare hereditary disorder currently identified in 200 families worldwide. It presents clinically with cutaneous, renal, and pulmonary manifestations due to an autosomal dominant mutation in a tumor suppressor gene, FLCN, on chromosome 17p11.2. We present a case of a 22-year-old male with Birt-Hogg-Dubé syndrome, showing typical features consistent with the classical type of EDS, with genetic testing revealing a COL5A1 mutation of "uncertain clinical significance", not yet reported in clinical literature. We discuss the treatment of this patient and describe the presentations of the two pathologies. Lastly, we put forth guidelines for the management of a dilated ascending aorta, with which this patient presents, for future patients who may present with this novel EDS mutation.

## Introduction

Ehlers-Danlos syndrome (EDS) is a set of genetic connective tissue disorders of variable inheritance characterized by joint hypermobility, skin hyperelasticity, and tissue fragility. Classification of connective tissue diseases began in the 1960s, and it wasn't until 1986 that EDS was officially classified, recognizing 11 subtypes based on the mode of inheritance and clinical features [1,2]. Since then, the nosology has been developed and refined into the currently used 2017 International Classification of the Ehlers-Danlos Syndromes, which lists 13 types of EDS [3].

The relevant type that will be discussed in this study will be classical EDS. Classical EDS is the most common form of EDS. In about 50% of patients with classical EDS, there is a mutation in two of the three genes for type V collagen, COL5A1 and COL5A2. Type V collagen is a minor collagen protein found in association with type I collagen in many tissues, such as skin, tendon, bone, cornea, placenta, and fetal membranes [4]. The COL5A1 gene is located at 9q34.2-q34.3 and is composed of 66 exons over >150 kb of gDNA, encoding the α1 chain of type V collagen [5]. Mutations in classical EDS are most often null mutations, resulting in haploinsufficiency [4]. Approximately one-third of individuals with classical EDS have nonsense or frameshift mutations, leading to the non-functional COL5A1 allele [6-9].

Due to collagen's prominent role and wide distribution in the body, EDS presents with multiple manifestations arising from different organ systems. Most notably known are EDS's cutaneous manifestations. Patients may present with skin hyperextensibility characterized by skin that is easily extended, beyond what would be considered normal, and snaps back to position. Scars may present with a classic "cigarette-paper-like" appearance. Patients with classical EDS may also have skin with a "velvety" texture, which is fragile and easily split with minor trauma [10]. Neurologically, patients with classical EDS may present with hypotonia, poor ambulation, delayed motor development, and mild motor disturbance. One feature of EDS is an abnormal capillary structure with a deficiency of surrounding collagen support, leading to the rupture of capillaries when subject to sheer force. Thus, patients may have easy bruising, presenting with spontaneous ecchymosis, creating a brown discoloration in areas such as the elbow and knees. Musculoskeletal manifestations of classical EDS consist of joint hypermobility, leading to articular complications, such as recurrent joint dislocations, chronic joint pain, foot deformities, temporomandibular dysfunction, and joint effusions. The most common areas affected are the shoulder, patella, digits, hip, radius, and clavicles. Patients may also experience vertebral abnormalities and decreased bone density [11]. Seldom, classical EDS presents with cardiovascular manifestations such as mitral or tricuspid valve prolapse and aortic root dilation. Rupture of large arteries occurs rarely with more severe and uncommon forms of classical EDS, but is most prominent in vascular EDS [5]. Reports of aortic root dilation in patients with classical EDS are becoming more common, with a higher prevalence in younger populations [12-14].

Birt-Hogg-Dubé syndrome (BHD) is a hereditary disorder, identified in only 200 families worldwide, and has been identified to be an autosomal dominant mutation in the FLCN gene. The FLCN gene locus was found to be located on the 17p11.2 chromosome by linkage analysis and codes for the protein folliculin [15,16]. It has been established that FLCN functions as a tumor suppressor gene. The proposed mechanism by which folliculin works is by negative regulation of the mechanistic target of rapamycin (mTOR), a protein that controls cell growth and protein synthesis. Insertions/deletions, nonsense, and splice-site mutations in FLCN lead to premature truncation of the folliculin protein, causing loss of function [17].

The clinical manifestations of Birt-Hogg-Dubé syndrome are thus far documented to affect the kidneys, lungs, and skin. Cutaneous symptoms usually appear after the age of 20 and consist of multiple, dome-shaped papules on the face, mainly on the nose and cheeks. These lesions are designated as fibrofolliculomas based on their histology [15]. BHD may also present with multiple facial angiofibromas [18]. BHD is known to be associated with renal cancer, with some studies showing renal tumors in 27% of individuals with BHD with a mean age of 50.5 years and other studies showing a prevalence of clear-cell renal cancer [19-21]. Pulmonary manifestations of BHD typically present with lung cysts, typically in the basal lung regions, with preserved lung function. On histology, this lung pathology is consistent with emphysematous changes [22-25]. One study reports a prevalence of spontaneous pneumothorax in 24% of patients with BHD at a median age of 38 years old [23].

## Case presentation

**Figure 1:**
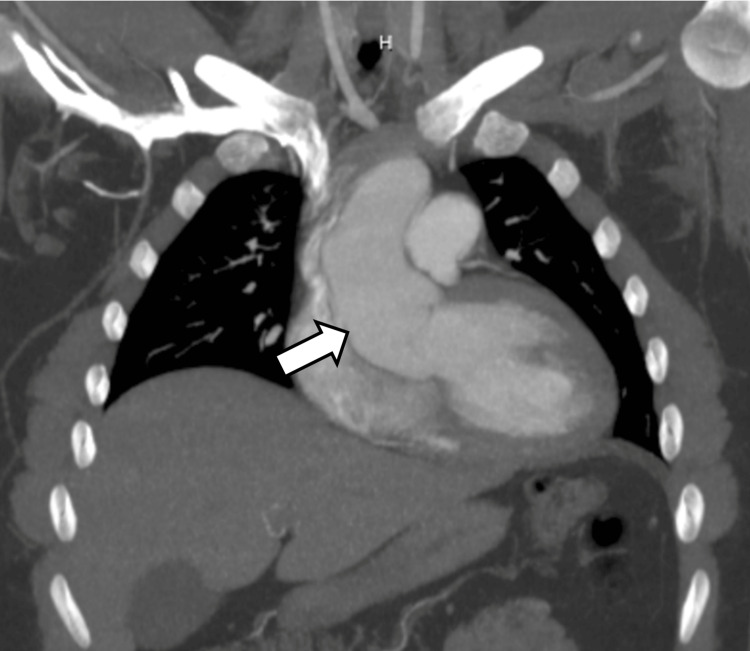
CT angiogram chest with contrast revealing a transverse diameter of the ascending aorta at the level of the aortic root at 4.6 cm

A 21-year-old male who had been previously under the care of cardiology at the local children's hospital was referred to the office for continuity of care in 2017. The patient has a history of congenital cardiac abnormalities such as patent ductus arteriosus, pulmonic stenosis, bicuspid aortic valve (BAV) with some stenosis, and dilation of the ascending aorta. The patient also has a history of Hashimoto's disease and hypertension. Due to the patient's history, the patient returned to the office for an echocardiogram. The echocardiogram revealed a bicuspid aortic valve, mild aortic root dilation of 4.6 cm, mild left atrial enlargement, estimated ejection fraction of 65%, trace mitral valve regurgitation, trace tricuspid regurgitation, and trace aortic valve regurgitation, with the rest of the study being normal. The echocardiogram was repeated annually with no significant changes. The patient was started on 2.5 mg nebivolol PO daily for the management of the ascending aorta dilation (Figure 1). The patient will be continued to be evaluated through an echocardiogram annually to monitor changes in the dilation of the ascending aorta.

**Figure 2:**
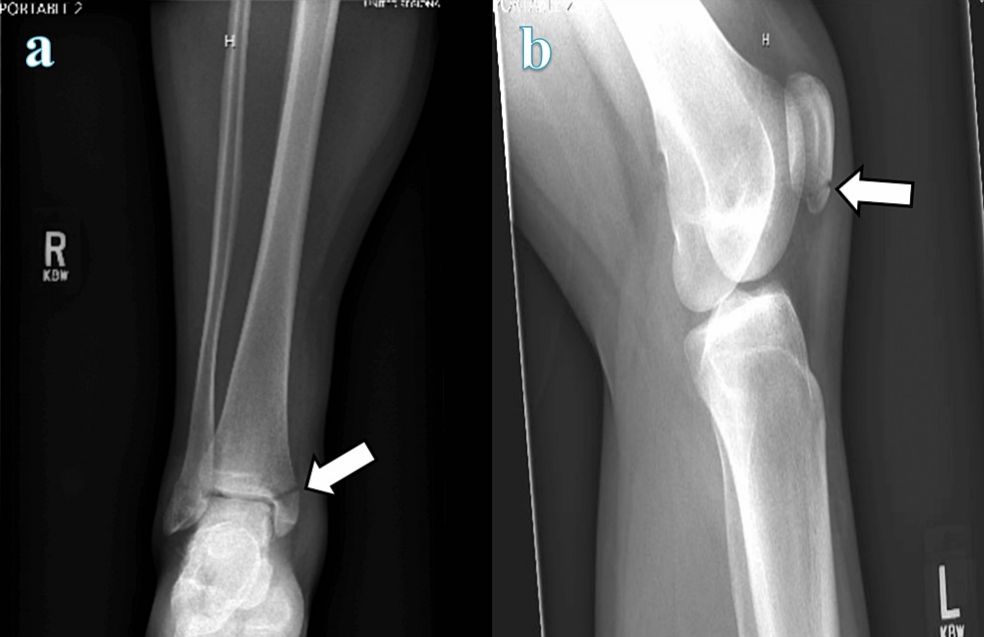
a) Traumatic bimalleolar fractures; b) Acute traumatic fracture involving the lower aspect of the body of the patella

**Figure 3:**
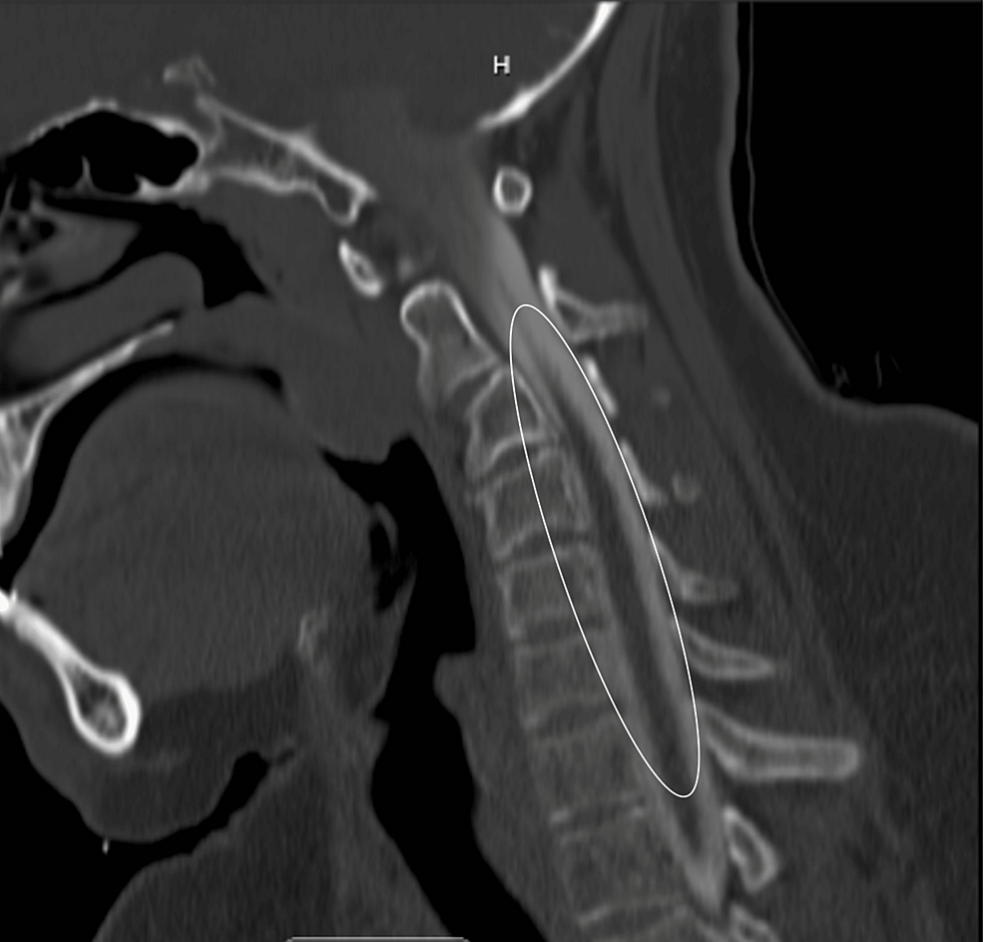
CT cervical myelogram with mild disc bulge present from C3-C7 with no foraminal stenosis

**Figure 4:**
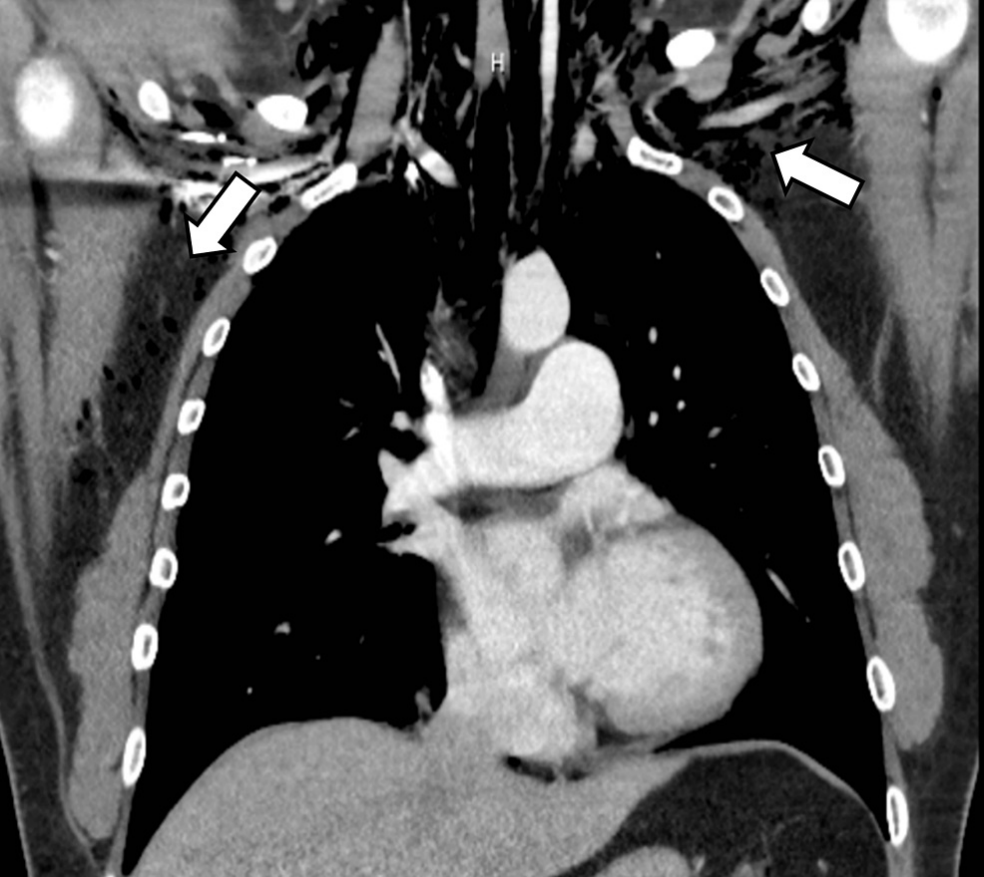
Chest CT with contrast showing extensive pneumomediastinum and subcutaneous air present in the chest wall and base of the neck. There are also bilateral apical pneumothoraces. The findings are consistent with the rupture of a small airway

The patient has reported the incidence of multiple fractures occurring from minor traumas throughout his life. The patient states he has sustained a fracture to the left patella, left wrist, left elbow, right ankle, and right wrist (Figure 2). These injuries occurred from falls from standing and from one minor motor vehicle accident (MVA). The patient also has a history of chronic neck pain with slight paresthesia in the upper extremities. A CT cervical spine myelogram was performed which revealed multiple bulging discs from C3-C7 (Figure 3). On physical examination, there are multiple fibrofolliculomas scattered diffusely around the cheeks and neck. In 2018, while working as a guard in the county jail, the patient was exposed to a significant amount of pepper spray, which led to the development of subcutaneous emphysema from a small pneumothorax leak, confirmed by chest CT (Figure 4). This unusual constellation of symptoms consisting of multiple fractures, skin lesions, and spontaneous pneumothorax warranted genetic testing to reveal a possible underlying cause. Per full gene analysis, there was found to be a positive pathogenic c.1285delC (g.17119709) in the FLCN gene, consistent with the diagnosis of Birt-Hogg-Dubé syndrome. An aortopathy panel was performed and revealed the presence of a heterozygous nucleic acid change of c.3939G>T in the COL5A1 gene with uncertain clinical significance. This variant has not yet been reported in medical literature but is currently reported in ClinVar (Variation ID: 365725).

The patient states his mother has a positive history of Birt-Hogg-Dubé syndrome with two spontaneous pneumothoraces, blebs in the pleural cavity, facial folliculomas, and cysts on the kidneys, sinuses, ovaries, and breasts. With a confirmed diagnosis of BHD through genetic testing and a positive family history, a renal ultrasound was performed to rule out renal cysts, which were found to be normal.

## Discussion

Birt-Hogg-Dubé syndrome has been identified in 200 families worldwide, and although the penetrance of folliculin mutations in affected families is high, the presence of the cutaneous, renal, and pulmonary manifestations can vary [16]. The variability of presentation is evident in this patient. As stated, the mother presented with cutaneous, pulmonary, and renal symptoms, whereas the patient did not have any renal cysts per ultrasound. This variance in presentation could also be due to the age of the patient, as current literature records the current onset of renal manifestations of BHD occurring at an average age of 50 years (30-70 years old) [19-22]. Therefore, this patient and other patients who have not yet presented with a renal tumor require lifelong surveillance. There is currently no consensus on surveillance guidelines, but it has been proposed that surveillance starts at 21 years of age and is repeated every 36 months, with MRI as the choice of imaging to reduce radiation exposure, and since ultrasound may miss small renal masses [26,27].

Genetic testing in this patient revealed a COL5A1 mutation of uncertain clinical significance, only reported three times before in ClinVar, with no documentation in any medical literature. We propose that mutation may be pathologic due to this patient presenting with manifestations consistent with the classical type of EDS. Specifically, this patient presents with musculoskeletal and vascular signs associated with EDS. It has been documented throughout clinical literature that patients with EDS may present with decreased bone density and vertebral abnormalities [11]. Our patient presents with a history of multiple fractures, which occurred due to low-velocity traumas, and has a history of chronic neck pain associated with bilateral upper extremity paresthesia. These symptoms are not better explained by any other pathology and, therefore, could be caused by this COL5A1 mutation. Secondly, the presence of aortic root dilation in patients with classical EDS has been documented to be as high as 6% [12-14]. This could explain the presence of the 4.6 cm dilated aortic root in our patient. The presence of these symptoms and the lack of other classical EDS symptoms could be due to the unique presentation of this specific mutation or could be due to the variable expressivity. 

A thoracic aortic aneurysm (TAA) can arise from multiple etiologies but arose in this patient due to EDS. TAA is usually asymptomatic but should be monitored closely in order to prevent complications, such as dissection and rupture. Lifestyle modification, such as smoking cessation, is important in mitigating adverse events of TAA [28]. Cigarette smoking is associated with the expansion of TAA and poses a higher risk for rupture. Smoking also causes degenerative changes in the mechanical properties of the vessels [29]. Anti-hypertensive therapy with beta-blockers should be initiated, with a goal of systolic blood pressure of 105 to 120 mmHg, to inhibit further aortic expansion. ACE inhibitors and angiotensin receptor blockers can also be used if the beta-blocker is not well tolerated [30,31]. Although there is a significant portion of patients with classical and other types of EDS, excluding vascular-type, with aortic root dilation, the progression to dissection is rare [23]. Still, compared to the general population, this population is at a greater risk for complications of aortic root dilation. This patient is also at greater risk due to the presence of a bicuspid aortic valve. According to the American College of Cardiology (ACC), with a strong (class 1) strength of recommendation, patients such as this with a diameter of the aortic root ≥4.0 cm require lifelong surveillance of the aortic root and ascending aorta by a transthoracic echocardiogram (TTE), CT, or MRI, at a rate dependent on the size of the aortic diameter and rate of growth. The ACC also recommends, with moderate (class 2a) strength of recommendation, that patients with BAV with a diameter of 5.0 to 5.4 cm of the aortic root should undergo repair of the aortic root, ascending aorta, or both if an additional risk factor for aortic dissection is present. This additional risk factor in this patient being a "root phenotype" aortopathy [32].

## Conclusions

This patient presented with manifestations consistent with classical EDS with a novel mutation in the COL5A1 gene not yet reported in the medical literature. Therefore, we proposed that this mutation could have some clinical significance as this patient did present with symptoms of EDS, not better explained by anything else. This patient also has a rare disorder known as Birt-Hogg-Dubé syndrome. Currently, this patient only presents with cutaneous and pulmonary symptoms of BHD but will continue to be monitored for the possible appearance of renal cysts. Lastly, we presented guidelines for the management of aortic dilation in patients with this presentation, consistent with ACC guidelines, which will be followed in the care of this patient.
